# Influence of human capital, urbanization, fuel imports and other macroeconomic factors on electric vehicle adoption

**DOI:** 10.1016/j.heliyon.2025.e42661

**Published:** 2025-02-14

**Authors:** Judit T. Kiss, István W. Árpád, Dénes Kocsis

**Affiliations:** a Department of Engineering Management and Enterprise, Faculty of Engineering, University of Debrecen, 4032, Debrecen, Hungary; b Department of Mechanical Engineering, Faculty of Engineering, University of Debrecen, 4032, Debrecen, Hungary; c Department of Environmental Engineering, Faculty of Engineering, University of Debrecen, 4032, Debrecen, Hungary

**Keywords:** Electric vehicle, Human capital, Fuel imports, Urbanisation, Fixed-effect model

## Abstract

Given that the transport sector is one of the largest emitters of CO₂, promoting the growth of electric vehicles is an option in the fight against climate change, and individual policies need to develop appropriate measures. The study examined the factors influencing the spread of electric passenger cars using panel data from 2012 to 2021 in 18 European countries. Electricity prices, urbanization, fuel imports, real GDP per capita, human capital and household saving rate were included in the multivariable non-linear model. To the best of our knowledge, there is no previous study on the impact of household savings and the inclusion of these variables in one model for the sample of selected countries and periods. Based on the results, human capital, household savings, and urbanization have a significant positive effect on the spread of electric cars, while fuel import has a significant negative effect. The biggest impact was found on urbanization and human capital. Reducing dependence on fuel imports can positively affect the uptake of electric vehicles. At the same time, providing information on the environmental impact of electric vehicles and policies that support investment in human capital can also have a favourable indirect effect on the uptake of electric vehicles.

## 1 Introduction

Transport is one of the sectors that contributes significantly to greenhouse gas emissions. An important step is the market introduction of electric vehicles, especially those powered by renewable energy, and the increase of their traffic share to reduce environmental, air and noise pollution, as well as the emission of greenhouse gases. Global CO₂ emissions from the world's transport sector increased gradually between 1990 and 2019, after a temporary decrease until 2020 due to the pandemic, increasing again by 7 % in 2021. Global CO₂ emissions by 2021 were 66 % higher than in 1990 [1]. Within the European Union, carbon dioxide emissions from the transport sector increased by 16 % between 1990 and 2021 [1]. Between 1990 and 2019, the level of CO₂ emissions increased in most of the countries included in the current study, with the most significant increase in the Czech Republic (by 172 %) and Poland (by 218 %), but a decrease was observed in Finland (by 5 %), as well as in Sweden (by 18 %) [2]. The results of policies to reduce harmful emissions from the transport sector are visible, as a 10 % reduction in 2021 compared to 2005 was achieved in the European Union [1]. In the European Union, the proportion of passenger vehicles with electric motors compared to the total passenger car stock did not reach 1 % in 2021. The European Union ratio increased from 0.024 % (2013) by 0.74 percentage points to 0.76 % by 2021 [3]. The Netherlands, Denmark and Sweden had the highest vehicles with electric motors rates at 2.78 %, 2.39 % and 2.21 %, respectively, in 2021 [3]. The market introduction of electric vehicles, which also depends on the nature of the charging infrastructure and the high price of electric passenger cars, rose the least between 2013 and 2021 in Cyprus (by 0.055 percentage points) and Poland (by 0.058 percentage points) and reached 0.056 % and 0.07 % by 2021, respectively. From the point of view of climate and energy policy, as well as transport policy decisions and the taking of the necessary measures, a priority area is the forecasting of the number of electric vehicles, the examination of the factors influencing their market demand, and the examination of the impact of the introduction of electric vehicles on the environment and the economy.

Several studies have addressed the increased market penetration and demand for electric vehicles [[4], [5], [6], [7], [8], [9], [10], [11], [12], [13], [14]]. The studies can be grouped according to whether they focus on vehicle characteristics, such as price, lifetime, charging time, socio-psychological tests (environmental awareness or drive preferences) or economic factors. One part of the economic studies falls under the scope of macroeconomic studies, within which they are limited to the investigation of factors influencing the market demand for electric vehicles, such as GDP, urbanization, renewable energy production, financial purchasing incentives, unemployment, charging opportunities for different countries, groups of countries and using various methodologies [7,8,11,12,[15], [16], [17], [18],^65^].

The other part of the micro-level economic studies examines the consumers' behaviour and decisions to reveal the main factors of electric vehicle consumers' demand [13,[59], [60], [61], [62]].

Long-term time series and panel data for electric vehicles were not yet available, so previous research has been limited to a shorter period. Few studies have investigated the impact of human capital, fuel imports, savings rates and electricity prices on the market demand for electric vehicles, partly due to limited data availability [8]. Increasing the number of electric vehicles can reduce noise and pollution from traffic, and identifying the factors influencing the market demand for electric vehicles can help develop appropriate policies to increase the demand for electric vehicles. Analyzing the factors influencing the uptake of electric vehicles is particularly relevant as data has now been available for a longer period on which to base the analysis. In recent years, the uptake of electric vehicles has accelerated significantly in some European countries, while in others, the process has been slower. These differences are now well represented in the available data and can be analyzed. Looking at the more extended periods and the differences between countries allows more precise conclusions to be drawn about the economic, social and infrastructural factors influencing the uptake of electric vehicles. Analyzing the factors influencing the uptake of electric vehicles can be linked to policy issues such as combating climate change, ensuring sustainable economic growth, increasing energy independence and decarbonizing the transport sector. These issues are fundamental to national and international climate policies and economic policy goals focusing on the green economy and sustainable development. The spread of electric vehicles can be one of the means to achieve these political objectives. Therefore, thoroughly examining the factors influencing the spread can help policy decision-making.

The study belongs to the study group of macroeconomic factors influencing the adoption of electric vehicles. The purpose of the study is twofold. On the one hand, it supplements previous, shorter-term studies with the newly available data. On the other hand, the study aims to explore whether urbanization and the positive effect of investing in human capital on the economy also apply to adopting electric vehicles. The study can support identifying factors contributing to the rise of electric vehicles and help develop appropriate political incentives.

This paper examines the impact of human capital, urbanization, fuel imports, GDP, electricity prices, and household savings on the share of electric vehicles in 18 European countries between 2012 and 2021. In addition to Switzerland and Norway, all European Union member states participated in the study, for which data were available for the period under study.

This paper contributes to the literature by examining the impact of factors such as fuel imports and household savings, which are less discussed in the literature, on the uptake of electric vehicles. The effect of factors such as human capital, urbanization, energy imports and electricity prices are rarely examined comprehensively in a single study. The study is based on a sample of European countries that show significant differences in the uptake and evolution of EVs over time. To the best of our knowledge, a similar investigation has not yet been conducted in a selected sample of European countries included in the study. The paper is organized as follows: The second part of this study provides a literature review for the relevant research, the third part presents data and the methodology, and the fourth part shows the estimation results and the main findings. Finally, the article ends with conclusions.

## 2 Literature review

One of the objectives of the European Green Agreement is to reduce greenhouse gas emissions from transport by 90 % so that the EU can become a climate-neutral economy by 2050 [19]. Greenhouse gas emissions from the transport sector account for a quarter of the EU's total emissions and are the main source of air pollution in urban areas [19]. One of the main milestones of the green energy plan is that by 2030, at least 30 million zero-emission passenger cars and by 2050, almost all passenger cars will be zero-emission [19]. Research that reveals the determining factors of market demand for alternative fuel vehicles to develop suitable policy measures is essential.

The CO₂ emissions of electric vehicles can be influenced by vehicle-specific [20] and individual-specific socio-economic factors. Vehicle-specific factors include, among other things, the technology used in the production of batteries, battery lifetime, propulsion mode, and the EV stock and the nature of the energy source required for the operation of the vehicles, i.e., whether the energy comes from a renewable energy source [[21], [22], [23], [24], [25], [26], [27], [28]]. At the same time, from an individual-specific point of view, CO₂ emissions are also influenced by charging behaviour [29] or driving patterns [25]. Research confirmed that the increment in electric vehicle stock [[21], [22], [23],^27^] or operating EVs in a suitable environment, such as next to the intensive decarbonization of the electricity sector, could mitigate CO₂ emissions [30].

Factors influencing the demand for EVs can be grouped according to several aspects, which can be socio-economic factors (financial incentives, energy tax, unemployment, education, economic development, urbanization rate, population density, fuel prices, renewable energy production, fuel imports), socio-psychological factors (environmental activity, environmental concern, environmental awareness and attitude), or factors are more related to the technical characteristics of the vehicles (charging time, battery life, the distance that can be covered on one charge, fast-charge, top speed, efficiency).

Empirical research revealed that the demand for electric vehicles is positively influenced by the GDP per capita or the change in GDP [7,9,11,12,17], increase in the proportion of renewable energy production [7,12], financial purchase incentives [5,6,11,13,16,18,24], and the decrease in the number of unemployed people [5,12]. Using data from ten countries, a researcher concluded that GDP per capita, the availability of chargers, and the amount of renewable energy production positively influence EVs' demand [17]. At the same time, the effect of fuel prices was unclear, given that the examination resulted in different impacts of fuel price for two types of EVs (positive for the BEV and negative for the PHEV). Previous research has revealed that the price of EVs is an important factor affecting demand. Therefore, fiscal purchase incentives also have a positive effect on purchase decisions. Alali et al. examined the moderating effect of GDP on the electric vehicle market through financial incentives [11]. Their studies showed that GDP positively moderates the impact of financial incentives on the demand for electric vehicles. A study of 33 European countries covering 2009 and 2019 showed that GDP significantly negatively affects PEV market share [67]. Some studies assessed the impact of financial incentives on EV adoption, and mostly positive effects have been identified [6,7,52,54]. However, these findings show no clear evidence of the supporting implications of fiscal incentives, and more examinations are required. Although some studies have shown a positive impact of financial incentives on EV adoption, at the same time, the effect did not prove to be strong enough to build EV purchasing supported government policy on financial incentives [6].

In the United States, government financial incentives introduced by the new Energy Policy Act of 2005 increased the sales of hybrid electric vehicles from 3 % to 20 % depending on the model considered, but the incentive effect studied was effective when the amount provided was sufficiently large [5]. Other government measures can positively impact EV penetration, such as the charging infrastructure development or free parking possibilities for EV vehicles [31], free charging or access to bus lanes [13,32].

Financial savings associated with vehicle operation and individuals' higher income are positively related to electric vehicle adoption [72]. We are not aware of any empirical literature that has previously analyzed the impact of household savings on the adoption of electricity in addition to financial incentives and household income. It is assumed that households with higher savings will find it easier to cover the initial costs of buying an electric vehicle and more accessible to take out a loan if needed to purchase a vehicle. Electric vehicles' maintenance and running costs (e.g., cheaper fuel, maintenance and taxes) can lead to savings over time compared to conventional vehicles. We suppose households with higher savings have a longer-term perspective and are more inclined to make long-term beneficial investments. Hypothesis 1: Household savings are positively associated with adopting electric vehicles.

Social-psychological factors include individuals' environmental attitudes, which may influence individuals' environmentally conscious actions and may differ between educated and less educated individuals. Examining human capital as a socio-economic indicator can reveal whether the level of human capital affects the spread of electric vehicles. In some research, the effect of education is examined as an independent variable, but not in all cases has a significant positive impact been demonstrated [6]. A study on the sale of hybrid and electric vehicles in Mexico revealed a significant positive effect of GDP per capita on electric vehicle sales while a negative impact on the cost of electricity [9]. At the same time, a negative coefficient was obtained for the variable of educational development included in the model, but the effect was not significant [9].

Researchers analyzed the impact of renewable electricity production, charging density, gas price, education, GDP, urbanization rate and population density on electric vehicle demand for fourteen countries from 2010 to 2015 [7]. Based on the estimation results of the fixed-effect model, all variables showed a positive effect on EV demand, except for GDP, which had a negative and insignificant impact. Next to the GDP, gas prices and urbanization failed to show a significant effect [7]. An education attainment variable approximated by the percentage of college-educated adults aged 25 to 64 showed a significant positive effect on EV demand [7]. Their interpretation is that if the education variable increases by 1 %, EV demand can increase by 19 %, and all else is held constant. The countries included in the study accounted for more than 96 % of global sales, indicating that the proportion of EVs was significantly higher in these countries than in others. Further investigation is needed to determine whether the revealed effects can also be generalized to countries with a lower rate of EVs.

We hypothesize that the EV's ratio in the total vehicle stock is higher in countries with higher human capital. Hypothesis 2: Human capital is positively associated with the demand for electric vehicles.

From the point of view of transport policy, it is also crucial whether the density of charging stations influences the adoption of EVs. One study highlighted the significant positive impact of the availability of charging stations on the demand for EVs [6].

Studies investigating the effect of population density have shown that it positively affects the demand for electric vehicles [7,17,53,54], while other studies explored a negative relationship [12]. To our knowledge, few studies have examined the impact of human capital and urbanization on EVs' market share. The studies that included the proportion of the urban population in their models reported both negative [66] and positive significant [67] as well as non-significant effects [7]. A study revealed a significant negative impact on EVs, supporting the research hypothesis that BEVs tend to be bought by people living in rural areas or suburbs [66]. Research related to Europe showed that the proportion of the urban population positively affects the PEV (Plug-in Electric Vehicle) market share, concluding that despite limited charging options in dense urban centres, with greater urbanization, the range-limited PEV ratio is greater [67].

The integration of urbanization into the model stems from considering that developing a greener urban policy can contribute to promoting urban electromobilization. The urban population with a higher average level of education and income may prefer electric vehicles for shorter distances in urban transport, which results in lower noise and CO2 emissions, especially if the infrastructure supporting the transport is adequately built.

Transitioning urban public transport systems to electric vehicles can reduce harmful emissions and create a cleaner, more livable urban environment. Integrating electric vehicles into public transport, such as taxi fleets, can reduce the total cost of urban transport by 0.2 %, demonstrating economic viability [85].

According to our assumption, urbanization positively affects the spread of electric vehicles in the European countries chosen as a sample. Hypothesis 3: Urbanization positively affects the spread of electric vehicles.

In several countries, importing fuel to run conventional vehicles is significant. High fuel imports make the balance of payments vulnerable to oil price shocks; however, if electricity is generated from renewable energy or even other domestic fossil fuels, electric mobility can positively impact energy security and the macro-economy [74]. The high fuel import dependency and the country's exposure to oil price fluctuations can be reduced by increasing EV' adoption. A study for the UK showed that if the fuel supply of vehicles is diverted from the more import-intensive gasoline and diesel to the output of the domestic electricity industry with electromobility, it has a multiplier effect on the growth of the economy [73]. In several countries with high populations, such as China, India and the USA, oil import dependence is very high, which makes it difficult to reduce CO2 emissions related to transport. In the European Union, import dependence reached 97.7 % in 2022 [75].

In line with the assumptions, the reduction of dependence on fuel imports has a positive impact on the market for electric vehicles. H4: Fuel import dependence is negatively related to the adoption of electric vehicles.

Given that the research found in the literature does not support the significant influence of several socio-economic indicators on demand for electric vehicles in many cases, further research is vital for developing appropriate government policy. On the other hand, although the research methodology and the scope of the examined countries are different, the researchers were able to cover a shorter period to analyze the adaptation of electric vehicles in their research. In addition to expanding the time series data for electric vehicles, new research is essential to reveal more precise connections.

## 3 Data and methodology

### 3.1 Data explanation

Panel data for 18 European countries with seven variables were involved in this study. The countries concerned are Austria, Belgium, Czechia, Denmark, Estonia, Finland, Germany, Hungary, Ireland, Italy, Latvia, Luxembourg, Norway, Poland, Portugal, Spain, Sweden and Switzerland. The study period covers 2012 to 2021, during which data were available for most European countries studied. The dependent variable is the number of electric passenger cars (EV) as a percentage of total passenger cars. Data for electric vehicles, excluding plug-in hybrids and hybrid electric vehicles, were available from 2012. The independent variables include electricity prices (EP), the urban population as a % of the total population (UP), fuel imports (FI), real GDP per capita, human capital and household saving rate (HS). Several indicators are used in the empirical literature to approximate education or human capital as an independent variable, such as the human capital index [[33], [34], [35]], average years of schooling [36,37], the ratio of individuals with tertiary education level or at least secondary education level as a percentage of the 15–64 age group [6,38], and secondary school enrollment rate [36]. Human capital (HC) is measured in this study as a percentage of individuals with completed tertiary education. Data for fuel imports and urban population were collected from the World Bank database [39,40], and the other annual data were obtained from the Eurostat database [3,[41], [42], [43], [44]]. The summary of the data involved in the panel model estimation is listed in Table 1.

**Table 1:** Variables of the model and their sources.

Variable	Data	Sources
EV	Number of electric passenger cars using an electric motor as a percentage of total number of passenger cars (%)	Eurostat [3]
EP	Electricity prices for Medium size households (euro)	Eurostat [41]
UP	Urban population as a % of the total population	World Bank [39]
FI	Fuel imports (% of merchandise imports)	World Bank [40]
GDP	Real GDP per capita in EUR 2010	Eurostat [42]
HC	Percentage of individuals with completed tertiary education (levels 5–8) as a percentage of the 15–64 age group % (working age)	Eurostat [43]
HS	Gross household saving rate	Eurostat [44]

### 3.2 Model specification

The main goal of this analysis is to explore the relationship between electricity price, human capital, GDP, urban population, fuel imports, household saving rate and electric vehicle penetration. The panel model specification is as follows:

$$y_{it}=\alpha_{i}+{\beta^{\prime }X}_{it}+\varepsilon_{it}\text{,}$$

where *i* and *t* stand for the country and year, $X_{it}$ represents the independent variables for *i*-th country and *t*-th year, $\varepsilon_{it}$ is the error term. The model estimation assumes different intercepts between cross-sections. The assumed economic model with the six independent variables is as follows:

EV=f(EP,UP,FI,HC,HS,GDP)

Assuming a non-linear model, we took the logarithm of the dependent and independent variables to reduce heteroskedasticity. The specified log-linear fixed-effect model (FEM) model inserted the dependent and independent variables is shown:

$$\ln\;Y_{it}=\beta_{0}+\beta_{1}\;\ln\;{EP}_{it}+\beta_{2}\;\ln\;{UP}_{it}+\beta_{3}\;\ln\;{FI}_{it}+\beta_{4}\;\ln\;{HC}_{it}+\beta_{5}\;\ln\;{HS}_{it}+\beta_{6}\;\ln\;{GDP}_{it}+\varepsilon_{it}\text{,}$$

where $Y_{it}$ is the dependent variable represents the number of electric passenger cars as a percentage of the total number of electric passenger cars, ${EP}_{it}$ is the electricity price, ${UP}_{it}$ is urban population, ${FI}_{it}$ is fuel imports, ${HC}_{it}$ is human capital*,* ${HS}_{it}$ is the household saving rate and $GDP_{it}$ is the GDP per capita. $\beta_{1},\beta_{2}\ldots \beta_{6}$ are the parameters of the independent variables, $\varepsilon_{it}$ is the normally distributed error term. The coefficients of the independent variables show the elasticity indicator of the electric passenger cars indicator concerning the one percentage change for the given independent variable, which holds the others constant. The index *t* and *i* indicate country and time.

## 4 Results and discussion

### 4.1 Descriptive statistics and correlation

**Fig. 1:**
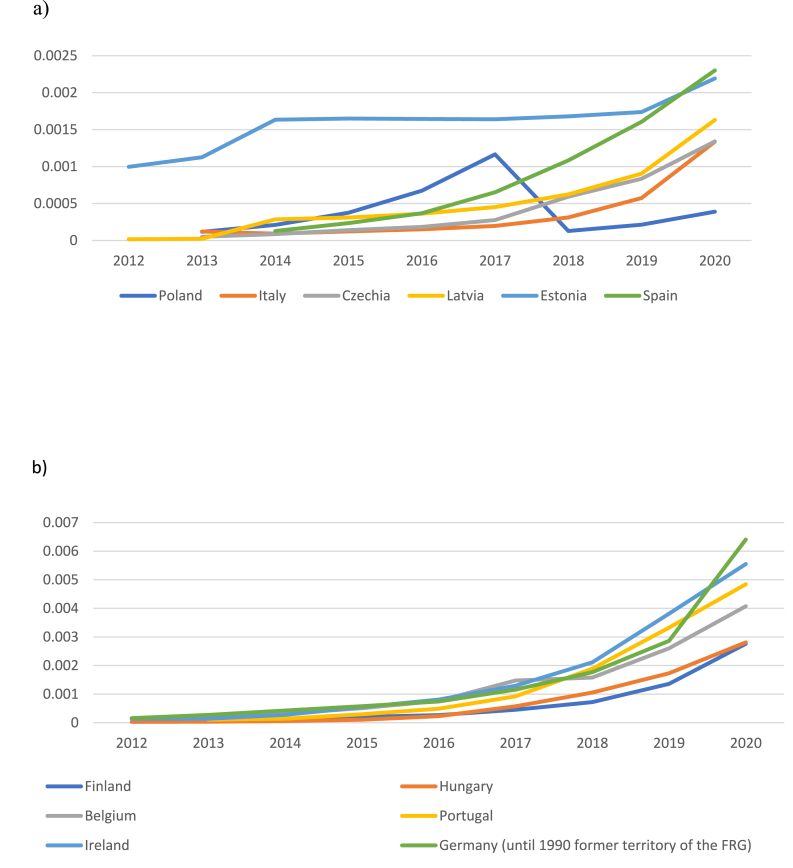

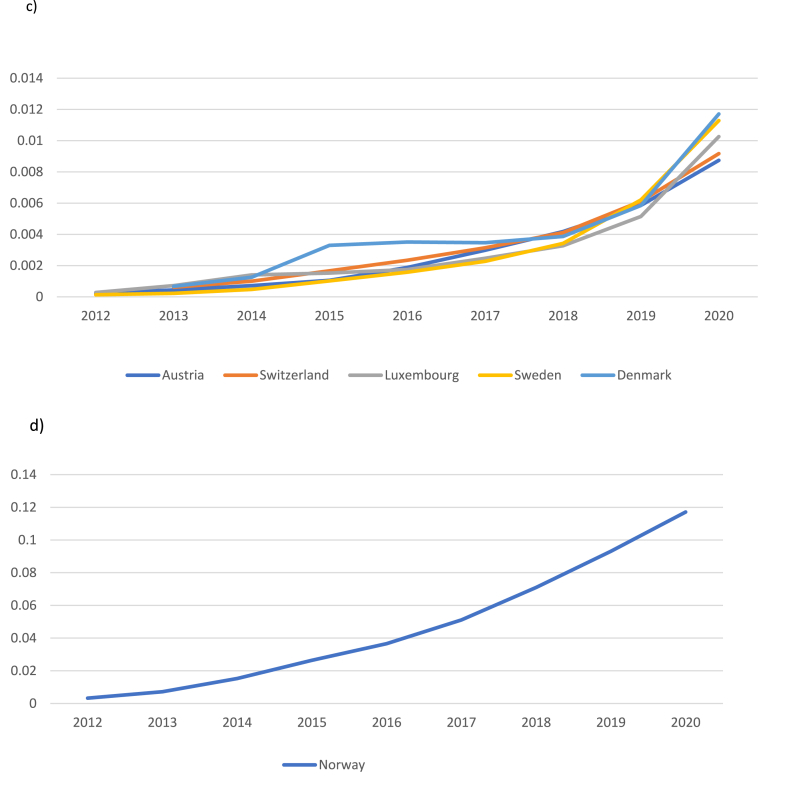
Number of electric passenger cars using an electric motor as a percentage of total (%) a) group of countries with values below 0.25 % in 2020 b) group of countries with values between 0.25 % and 0,65 % in 2020 c) group of countries with values between 0.65 % in 2020 and 1.2 % d). values for Norway. Source: [3].

**Fig. 2:**
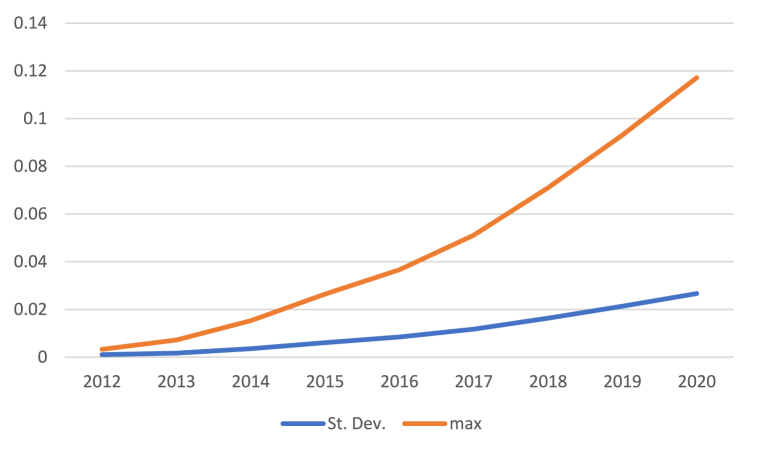
The standard deviation of the proportion of EVs (%) and the evolution of the highest value (%) over time for the entire sample. Source: [3].

The minimum and maximum values of the passenger electric vehicles as a percentage of total passenger cars (EV) are 0.0016 % (Latvia, 2012) and 15.5 % (Norway, 2021), while the mean is 0.57 % (Table 2, Fig. 1a–d, see in Appendix). Except for Norway, where the share of electric vehicles is the highest, countries can be divided into three groups according to how electric vehicle penetration will develop in 2020. In the three groups with different ranges, the countries are evenly distributed (those below 0.25 % - 6 countries, those between 0.25 % and 0.65 % - 6 countries, and those above 0.65 % - 5 countries), which illustrates the variability and time trends in the data and the need to examine the impact of factors influencing the data. In the sample, the proportion of EVs in the total vehicle stock gradually increased from 2013, but the growth rate accelerated significantly after 2017 (Fig. 2, see in Appendix). The percentage of electric vehicles and the change in standard deviation over time reflect that some countries have made much faster progress in adopting electric vehicles, while others have lagged (Figs. 1 and 2. see in Appendix). Based on the data of the countries included in the sample, it can be seen that the electric vehicle market is not homogeneous. It is essential to understand what is behind divergent development, for example, what economic and social factors help or hinder individual countries. The mean for electricity prices is 0,19 EUR, with a minimum of 0.06 EUR (Spain, 2015) and a maximum of 0.32 EUR (Germany, 2021) (Table 2). The mean is 75.37 % for the urban population as a percentage of the total population, with 57.15 % minimum (Austria, 2012) and 98.12 maximum value (Belgium, 2021). The mean fuel imports as a percentage of merchandise imports is 9.74 %, with a minimum of 2.08 % (Switzerland, 2020) and a maximum of 22.85 % (Finland, 2013). The countries with the highest fuel import rate, such as Finland and Belgium, significantly reduced their import dependence by 2021 and had a rate below 14 % by 2021. Next to the dependent variable, GDP is the indicator with the largest relative deviation, with the smallest value of 9680 EUR per capita (Latvia, 2012) and the most considerable value of 84750 EUR per capita (Luxembourg, 2016). The percentage of individuals with tertiary educational levels can be found between 14.4 % (Italy, 2013) and 45.2 % (Ireland, 2021), with a 30.46 % average. The percentage value of the household saving rate is 12.57 on average, with a minimum of −3.32 (Latvia, 2013) and a maximum value of 27.00 (Switzerland, 2020) (Table 2).

**Table 2:** Descriptive statistics (Obs 171).

Variable	Mean	Std. Dev.	Min	Max
EV	0.57	1.79	0.0016	15.5
EP	0.19	0.06	0.06	0.32
UP	75.37	10.94	57.15	98.12
FI	9.74	4.14	2.08	22.85
GDP	34430.99	21015.27	9680.00	84750.00
HC	30.46	7.21	14.40	45.20
HS	12.57	5.33	−3.32	27.00

Correlation coefficients were calculated to explore the relationship between the dependent and independent variables. Table 3 presents the coefficients among the variables. A positive and significant correlation exists between the number of electric passenger cars as a percentage of the total number of passenger cars (EV) and all other variables with at least a 5 % significance level except electricity price and fuel imports. The correlation between EV and electricity prices shows a negative, non-significant and low degree of association. At the same time, a negative correlation between fuel imports and electric vehicles can be detected at a significance level of one per cent. The largest association can be found between the real GDP per capita and human capital, with a value of 0.6284. The correlation coefficients between pairs of independent variables are in the acceptable range. Apart from the mentioned highest correlation value, the other absolute values are lower than 0.5, i.e. lower than the threshold value of 0.8.

**Table 3:** Correlation analysis.

Variable	EV	EP	UP	FI	GDP	HC	HS
EV	1.0000						
EP	−0.0046	1.0000					
UP	0.1598∗	0.1784∗	1.0000				
FI	−0.2600∗∗	−0.0907	0.0714	1.0000			
GDP	0.3688∗∗	0.2506∗∗	0.4695∗∗	−0.4179∗∗	1.0000		
HC	0.2825∗∗	−0.0405	0.4185∗∗	−0.2381∗∗	0.6284∗∗	1.0000	
HS	0.2134∗∗	0.2052∗∗	0.2320∗∗	−0.4767∗∗	0.4871∗∗	0.2608∗∗	1.0000

Note: ∗ and ∗∗ denote 5 % and 1 % significance levels, respectively.

### 4.2 Cross-sectional dependence (CSD)

Cross-sectional dependence was analyzed in two steps. First, the CSD test was analyzed for the variables. Based on the results in Table 4, the variables are cross-sectionally dependent. Second, the cross-sectional dependence test examines whether the error terms are independent across sections. Breusch and Pagan proposed the Lagrange multiplier test that can be applied to the panel models with a large value of T and a smaller value of N [46]. Pesaran suggested an alternative based on the pair-wise correlation coefficients, not the squares used in the LM test. The Pesaran's CSD test is more applicable for the panel data with large N and small T [47,48]:

$$CD=\sqrt{\frac{2T}{N\left(N-1\right)}}\sum_{i=1}^{N-1}\sum_{j=1+1}^{N}{\hat{\rho }}_{ij}\text{,}$$

**Table 4:** Cross-sectional dependence test result.

	EV	UP	FI	HC	HS	GDP	EP
CD-test	33.372	28.926	31.967	31.454	24.02	28.886	3.253
P-value	0.000	0.000	0.000	0.000	0.000	0.000	0.001

the CD test for the unbalanced model is as follows:

$$CD=\sqrt{\frac{2}{N\left(N-1\right)}}\sum_{i=1}^{N-1}\sum_{j=1+1}^{N}\sqrt{T_{ij}}{\hat{\rho }}_{ij}\text{,}$$

where ${\hat{\rho }}_{ij}$ is the pair-wise correlation of the residuals. The null hypothesis of the cross-sectional independence was rejected by at least a 1 % significance level (Table 5).

**Table 5:** Cross-sectional dependence test.

Test	Results	Prob.
Breusch-Pagan LM	424.203	0.0000
Pesaran	3.215	0.0013

### 4.3 Model selection

We regressed the number of electric passenger cars as a percentage of total passenger cars on the households' electricity prices, the urban population as a % of the total population, fuel imports, real GDP per capita, human capital and household saving rate. F and the Hausman tests were conducted to select the appropriate model between pooled, fixed, and random effect models (Table A1; Table 6) [45]. Based on the tests, the fixed effects model is preferred.

**Table 6:** Hausman test results.

	Chi-Sq.	Chi-Sq. d.f	Prob.
Cross-section random	15.00	6	0.0202

By using fixed-effect models, it is possible to check the characteristics of the examined cross-sections if these characteristics do not change over time, even if we do not have data for them [68]. Fixed effects models can control for unobserved heterogeneities. While some variables are complex to observe or for which we do not have data, the fixed effects model allows us to control for time-invariant omitted variables [71].

The parameters, their standard error and t statistics of the estimated log-linear fixed-effects model are presented in Table 7. Based on the estimation results (Table 7), each explanatory variable significantly affects the explained variable. Contrary to the results of previous research, which examined the effect of household electricity price [6,63,64] and our expectation, the electricity price for medium households positively affects the number of electric passenger cars as a percentage of the total number of passenger cars. One explanation could be that, in addition to the increase in electricity prices, fuel prices for traditional cars also rise in parallel, and thus, the purchase of an electric vehicle is even more favourable. Furthermore, in addition to higher electricity prices, there is a greater incentive to build household solar systems, and thus, the household electricity produced can be used to charge electric vehicles. The result is in line with the findings of a study that EV adoption is likely to increase if the difference between fuel and electricity prices increases [11]. Further examinations are needed to explain the relationship between the two variables and show the moderating effect of fuel prices on electricity prices.

**Table 7:** Results of the fixed-effects model regression analysis.

Variable	Coefficient	Std. error	t	Prob.
Cons.	−271.7147∗∗∗	29.9812	−9.06	0.000
EP	0.8432∗∗	0.3887	2.17	0.032
UP	54.4523∗∗∗	7.4412	7.32	0.000
FI	−0.9614∗∗∗	0.2322	−4.14	0.010
GDP	1.9527∗∗∗	0.7437	2.63	0.000
HC	3.7052∗∗∗	0.7916	4.68	0.005
HS	0.4346∗∗∗	0.1524	2.85	0.000

Notes: ∗, ∗∗ ∗∗∗ denote statistically significant at the 10 %, 5 %, and 1 % levels.

To test the reliability of the estimated model, we examined multicollinearity and heteroskedasticity. The multicollinearity was analyzed for all variables using the Variance Inflation Factor (VIF) and its reciprocal. Multicollinearity occurs when two or more explanatory variables in the model are highly correlated, limiting the reliable interpretation of the parameters. Table 8 shows the result of the multicollinearity test. Even though the highest value of multicollinearity can be detected for GDP, the multicollinearity indicators of all variables are well below the critical value, meaning there is no high degree of multicollinearity.

**Table 8:** Multicollinearity test.

Variable	VIF	1/VIF
EP	1.35	0.74
UP	1.55	0.65
FI	1.48	0.68
GDP	2.77	0.36
HC	1.79	0.56
HS	1.54	0.65

In the presence of heteroscedasticity, the variance of the errors of the regression model is not constant at all levels of the independent variable, which may result in biased estimates. To test for heteroscedasticity, we performed the Wald test, which shows heteroskedasticity (Table 9).

**Table 9:** Modified Wald test results.

Chi-Sq.	Prob.
410.96	0.000

The fixed effect models assume the homogeneity of the slopes. Testing for heterogeneous slopes is especially useful for large panels to avoid inconsistent conclusions. We tested the heterogeneity of the slopes using the heteroskedastic autocorrelation (HAC) robust estimator of Blomquist and Westerlund (Table 10) under the null hypothesis of homogeneity [69,70].

**Table 10:** The slope homogeneity test results.

	Delta	Prob.
	−0.393	0.694
adj.	−1.005	0.315

In the next step, we performed a robust fixed effect model estimation, considering that it provides robust standard errors and manages heteroscedasticity (Table 11).

**Table 11:** Results of the robust fixed-effects model regression analysis.

Variable	Coefficient	Robust Std. error	t	Prob.
Cons.	−271.7147∗∗∗	60.5405	−4.49	0.000
EP	0.8432	0.5128	1.64	0.118
UP	54.4523∗∗∗	14.5931	3.73	0.002
FI	−0.9614∗∗∗	0.2340	−4.11	0.001
GDP	1.9527	1.1348	1.72	0.103
HC	3.7052∗	1.5685	2.36	0.030
HS	0.4346∗∗∗	0.0997	4.36	0.000

Notes: ∗, ∗∗ ∗∗∗ denote statistically significant at the 10 %, 5 %, and 1 % levels.

### 4.4 Interpretation of results

The robust fixed-effect model estimation indicates no statistically significant relationship between electricity price and GDP and the adoption of electric vehicles (EVs). Concurrently, urbanization, human capital and household savings exert a significant positive influence, whereas fuel imports exert a significant negative influence on the penetration of electric cars. We might expect that in countries with a higher proportion of fuel imports, fuel supply and price predictability are more uncertain in addition to greater energy dependence, especially in extreme situations, increasing the demand for alternative vehicles. At the same time, if imports come from countries offering low fuel prices, the upkeep of electric vehicles will not be more cost-effective than that of conventional cars. Their demand will not increase unless the import of low-cost fuel is reduced. The finding is consistent with studies concluding that reduced dependence on fuel imports will promote the adoption of electric vehicles (EVs) [[76], [77], [78]]. Countries that rely heavily on fuel imports will probably be slower to switch to electric vehicles (EVs) due to the favourable price of imported fuel, economic dependence on imported fossil fuels, and the infrastructure that has been developed to accommodate these imports.

The coefficient for human capital is positive, representing that human capital has a positive statistically significant effect on the market share of electric passenger cars. Based on the estimated coefficient, if the percentage of individuals with completed tertiary education (of the 15–64 age group) increases by one percentage point, the electric passenger car share in the total number of passenger cars increases by 3.7 per cent if other influencing factors remain the same. The researchers came to a similar conclusion regarding the positive effect of human capital during the examination of the socio-economic factors of the demand for electric vehicles, but at the same time, in addition to human capital, they also showed a positive effect on urbanization, but the result was not significant [7]. An earlier survey for the European Union and the United States is also consistent with our research, according to which the market share of electric vehicles is higher in areas with a higher proportion of people with higher education [55]. When fitting education as an independent variable into the model, the variable is measured differently in different research, mainly with the proportion of people with a higher education, which has been shown to have a positive effect on the BEV (Battery Electric Vehicle) market share [[56], [57], [58]], but not on the PHEV (Plug-in Hybrid Electric Vehicles) [56]. Electrifying road transport can positively handle climate change and air and noise pollution. As has been previously documented in the literature, there is a positive correlation between the level of education attained by a given population and the rate of penetration of electric vehicles (EVs) [7,[55], [56], [57], [58],^80^,^81^]. Those with a higher level of education are more informed about environmental protection issues and more receptive to adopting new technologies, including electric vehicles (EVs).

In the literature, few studies have investigated the impact of urbanization, and no significant impact has been demonstrated [7]. Urbanization has the largest positive significant impact on the explained variable among the analyzed explanatory variables. A study similarly revealed a positive relationship between urbanization and PEV market share [67]. The estimation for the urbanization parameter represents that a 1 % increase in the urbanization rate increases the EV share in the total number of passenger vehicles by 54.5 %, holding other influencing factors constant. The result would mean that, e.g. in Austria, the EVs' ratio of 1.49 % (in 2021) would rise to 2.3 % if the urbanization rate increases by 1 %. The higher average level of education and income of the urban population, as well as the more favourable infrastructural background for the operation of EVs in urban areas, contribute to a wider uptake of electric vehicles. Several studies in the literature have also highlighted the positive relationship between EVs and urbanization [82,83].

In the same way, the GDP per capita has an insignificant impact on the market share of electric vehicles. Several studies have reached similar conclusions by fitting GDP per capita into the EV model [9,17]. An analysis of electric vehicles in a sample of 10 countries for 2013–2019 showed a positive effect of GDP per capita on the demand for electric vehicles [17].

Larger purchasing power has a positive effect on the demand for EVs. A one per cent increase in the gross household saving rate raises the dependent variable by 0.435 per cent if other influencing factors remain the same.

Households with higher savings can more easily cover the relatively high initial cost of buying an electric car and are also more likely to be able to install home chargers. Lower-income people buy mainly from the used electric car market, where supply is still limited [84]. At the same time, people with lower incomes are less able to accept the uncertainty associated with operating electric vehicles due to their lower risk tolerance. The government can help promote electric mobility by providing financial incentives and building a safe infrastructure for vehicles to operate.

## 5 Conclusions and policy implications

The impact of human capital, urbanization, fuel imports, electricity prices, GDP and household savings were examined on the demand for electric vehicles in 18 European countries between 2012 and 2021. Based on the Hausman test, the fixed effect model proved to be preferred. Using a fixed-effects model allows the research to control for country-specific, time-constant factors, ensuring the results can be more reliable. It is essential in analyses where countries have significant structural differences that could bias the estimates.

A more favourable economic environment, i.e., a higher GDP per capita and household savings ratio, positively impacts the demand for electric vehicles. The robust fixed-effects model estimation results show that the macroeconomic and socio-economic indicators involved in the model, the household saving rates, urbanization and human capital, significantly positively affect the dependent variable. The positive impact of GDP and electricity prices has not been proven significant.

Under the government's adequate environmental and energy policy, introducing and incentivizing measures to increase energy efficiency for the household sector can mean household savings and raise individuals' environmental awareness. Households with higher savings have greater purchasing power in the electric car market and are more likely to purchase electric vehicles with high one-off expenditures. Individuals with a higher propensity to save may be more responsive to the cost savings associated with operating electric cars, increasing EV penetration. For households with no or low levels of savings and those who cannot afford to live in cities but whose employment is limited to cities, the introduction of government financial incentives and well-structured tax on vehicle use can positively affect their demand for electric vehicles. By introducing purchase subsidies, such as low-interest loans (green credits) or tax incentives, and vehicle replacement programmes, governments can help to make electric vehicles more widely available. One of the primary impediments to the proliferation of electric vehicles (EVs) is the considerable upfront cost.

Consequently, policies that mitigate the high one-time expenses can facilitate the growth of the market [79]. Researchers came to a similar conclusion during their study on vehicle owners in Australia, according to which the application of appropriate financial incentives is essential in emission tax policy, given that taxing the use of vehicles either by a fuel tax or road pricing had the most significant impact on low-income households [49]. Beyond the impact of household income and financial incentives on the adoption of electric vehicles, no studies, to our knowledge, have determined whether the impact of household savings can be detected on EV rates.

In contrast to our expectations, the impact of fuel-related indicators on the market share of electric passenger cars is mixed. Fuel imports negatively affect the dependent variable, but the price of electricity has a positive but not significant impact. Reducing reliance on imported fuel sources and implementing policies promoting the use of low-carbon fuels can facilitate the wider adoption of electric vehicles (EVs) [79]. The positive effect of electricity prices for households on EVs can also arise from the point of view that households are less sensitive to the evolution of electricity prices, especially compared to the development of fuel prices; however, the assumption requires further investigation. The distance electric cars can cover on a full charge can be one of the main factors influencing the purchasing decision. Cars, used for short distances such as in urban areas, maybe more widespread if the share of the urban population is more extensive, as can be seen from the results obtained for the urban population share.

A study highlighted, among others, the role of human capital and its spillover effects from the point of view of environmental sustainability policy [50]. Our findings indicate that human capital, measured by the share of individuals with completed tertiary education as a percentage of the working-age population, has a statistically positive impact on the EVs' market share. The positive effect of human capital can be enhanced with a properly designed fiscal policy if it has an incentive force on investing in human capital, such as supporting higher education and providing tax incentives. Governments can create political incentives for the penetration of EVs, which can be direct, such as financial purchase incentives, and can be indirect, the effect of which is indirectly enforced, such as the investment in human capital. Individual environmental awareness and social responsibility can be promoted by providing information and education on the environmental impact of electric vehicles.

The analysis can contribute to cross-country comparisons and identify common factors important for EV uptake in each country. It is not only valuable for the scientific community but can also strengthen international cooperation in the promotion of electric vehicles.

The analysis can provide valuable information for policymakers in each country on which macroeconomic factors can help the uptake of electric vehicles, which can contribute to achieving sustainability goals. The novelty of the analysis can also be seen in the fact that it uses data combinations that have not been compared together before in the investigation of the spread of electric vehicles, such as human capital, energy imports and electricity prices. The examination covers a relatively short period due to the limited availability of the data, which indicates the limitations of our study. A similar problem is the limited availability of long time series for a comprehensive sample of countries related to electric vehicle transportation (e.g. fuel prices) and transport infrastructure (e.g., number of charging stations, charging prices). Therefore, the study was conducted for a small sample of countries. In the study, we did not examine some country-specific characteristics, such as financial incentives, subsidies, and tax burdens, the analysis of which requires further investigation. In the future, with the increase in the number of electric vehicles, the impact of the rise of electric vehicles on the economy can also be examined, as well as the effect on employment and tax revenues [51] and the causality with GDP and CO2 emissions. It is worth including the fuel price of conventional vehicles and the size of electricity imports as additional variables next to the electricity price in the model, reflecting whether a price effect exists.

## CRediT authorship contribution statement

**Judit T. Kiss:** Writing – review & editing, Writing – original draft, Methodology, Investigation, Formal analysis, Conceptualization. **István W. Árpád:** Writing – review & editing, Formal analysis, Conceptualization. **Dénes Kocsis:** Writing – review & editing, Formal analysis, Conceptualization.

## Data availability

Data will be made available on request.

## Declaration of competing interest

The authors declare that they have no known competing financial interests or personal relationships that could have appeared to influence the work reported in this paper.

## Figures and Tables

**Table A1:** Fixed-, random-effects and pooled-OLS estimation results.

Variable	Fixed effect	Random effects	Pooled OLS
Cons.	−271.7147∗∗∗	29.1104∗∗∗	−15.4350∗∗∗
EP	0.8432∗∗	0.8129	0.6535∗∗
UP	54.4523∗∗∗	1.3486	0.3029
FI	−0.9614∗∗∗	−1.4760∗∗∗	−1.126∗∗∗
GDP	1.9527∗∗∗	−0.6605	−0.0517
HC	3.7052∗∗∗	6.0167∗∗∗	2.8933∗∗∗
HS	0.4346∗∗∗	0.6165∗∗∗	0.7030∗∗∗
